# Measuring biological materials mechanics with atomic force microscopy ‐ Mechanical unfolding of biopolymers

**DOI:** 10.1002/jemt.24136

**Published:** 2022-05-02

**Authors:** Juan Carlos Gil‐Redondo, Andreas Weber, José L. Toca‐Herrera

**Affiliations:** ^1^ Institute of Biophysics, Department of Nanobiotechnology University of Natural Resources and Life Sciences Vienna (BOKU) Vienna Austria

**Keywords:** atomic force microscopy, energy landscape, I27, mechanical unfolding, proteins, thermal softening, titin

## Abstract

Biopolymers, such as polynucleotides, polypeptides and polysaccharides, are macromolecules that direct most of the functions in living beings. Studying the mechanical unfolding of biopolymers provides important information about their molecular elasticity and mechanical stability, as well as their energy landscape, which is especially important in proteins, since their three‐dimensional structure is essential for their correct activity. In this primer, we present how to study the mechanical properties of proteins with atomic force microscopy and how to obtain information about their stability and energetic landscape. In particular, we discuss the preparation of polyprotein constructs suitable for AFM single molecule force spectroscopy (SMFS), describe the parameters used in our force‐extension SMFS experiments and the models and equations employed in the analysis of the data. As a practical example, we show the effect of the temperature on the unfolding force, the distance to the transition state, the unfolding rate at zero force, the height of the transition state barrier, and the spring constant of the protein for a construct containing nine repeats of the I27 domain from the muscle protein titin.

**Highlights:**

1. Atomic force microscopy (AFM) can be used to study the mechanical unfolding of polymers.

2. AFM provides a direct measurement of unfolding (unbinding) forces.

3. Force measurements for different rates provide information about the distance to the transition state and the unfolding rate at zero force.

## Graphical abstract

Mechanical unfolding of (poly) proteins can be studied with atomic force microscopy. Above: sequential unfolding of the individual proteins until the (poly)protein is completely stretched and detached from the tip. Below: energy landscape that can be investigated with such experiments.

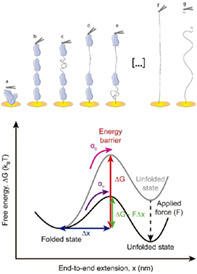

## 1 INTRODUCTION

### 1.1 Biopolymers and the importance of proteins

Biopolymers are carbon‐based chains of covalently linked monomers produced in organisms that cover crucial roles both inside the cell and in the extracellular matrix. Polynucleotides, DNA and RNA, constitute the blueprints used in the elaboration of the complex machinery present in all living cells. The complex machinery of enzymes, receptors, and scaffolds is mostly composed by another type of biopolymer: polypeptides or proteins. Another important type of biopolymers is polysaccharides, mostly not only involved in the storage of energy, but also implicated in the structural support of cells and tissues (Alberts et al., 2019).

Studying the mechanical unfolding of biopolymers provides important information about their mechanical stability as well as kinetics and energy landscape of folding and unfolding. This is especially important in proteins since their three‐dimensional structure is essential for their function. Proteins are polypeptides, flexible chains of amino acids linked by planar peptide bonds. They acquire a stable three‐dimensional structure due to the interactions between the backbone and side chains of the amino acids, including intramolecular interactions such as van der Waals, hydrogen bonds, electrostatic, and hydrophobic interactions and even covalent disulphide bonds. The unique shape of each protein determines its function, stability, and molecular targets. Some proteins act as enzymes that catalyze chemical reactions; other proteins bind to certain molecules and act as receptors; and others act as scaffolds inside and outside the cell (Hughes & Dougan, 2016; Stollar & Smith, 2020).

Almost every function of the cell depends on the correct folding and activity of proteins. Studying the different interactions that maintain the structure of each protein is important, since it allows us to understand its function and also how this function could be affected due to pathological changes in the structure of the protein. Classically, protein structure, folding and unfolding pathways have been studied by bulk denaturation methods, using chemicals (e.g., urea, alkaline, or acidic pH) or high temperature in combination with spectroscopic techniques (Almeida, 2016). Such methods sense different degrees of unfolding in the proteins, which give information about the folding and unfolding landscape, including the free energy of unfolding, the unfolding rate in the presence of denaturant and the distance to the transition state barrier. In a similar way, novel approaches are based on the unfolding of proteins using force. Single‐molecule force spectroscopy (SMFS) experiments involve the mechanical unfolding of a single protein in a specific direction. The main difference between SMFS and bulk denaturation with temperature or chemical reagents is that, in SMFS, the denaturing force is applied non‐isotropically on single macromolecules. This allows us to affect specific bonds inside the protein, depending on the direction of the force. Additionally, SMFS allows the study of differences between single molecules under the same conditions. Both bulk denaturation and SMFS can be used to obtain important information about protein structure and complement each other, but the mechanisms involved are different and comparisons between the information obtained through both methods should be carried out with caution (Carrion‐Vazquez et al., 1999; Fowler et al., 2002; Javadi et al., 2013; Stirnemann et al., 2014; Tapia‐Rojo et al., 2019).

Mechanical unfolding in SMFS experiments involve forces that range from tens of piconewtons up to few nanonewtons, which can be achieved through different techniques, including optical and magnetic tweezers, and atomic force microscopy (AFM) (Mora et al., 2020). In this primer, we will focus on the use of AFM.

### 1.2 Atomic force microscopy in SMFS experiments

**FIGURE 1:**
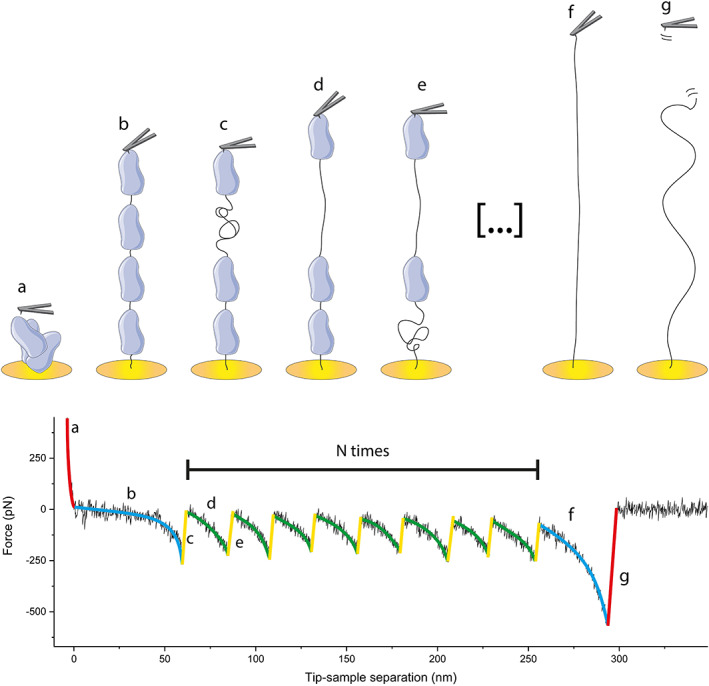
Unfolding of a polyprotein in a force‐extension AFM‐SMFS experiment. (a) The tip is first approached to the surface, and the protein construct unspecifically adheres to the tip. (b) The tip is retracted, the construct is straightened and the tensile force of the protein causes the cantilever to deflect toward the sample, which is registered as a (negative) increase in the force. (c) The pulling force overcomes the force that keeps one of the repeats in the construct folded, the repeat unfolds. This is registered as a sudden decrease in the force. (d) As the tip keeps pulling, the unfolded repeat straightens, which is seen as an increase in the distance between tip and sample and a gradual increment in the force caused by the resistance of the unfolded repeat to the straightening. (e) The pulling force overcomes the unfolding force of another repeat, which unfolds. This process repeats for a maximum of N times, being N the number of repeats of the POI present in the construct. (f) When the last repeat has unfolded, the whole construct is straightened, which is shown as a longer increment in the tip‐sample separation, until (g) The construct is finally detached from the surface or the tip, which normally requires a higher force. The nature of the process is probabilistic and the protein that resists less force unfolds first.

AFM is a useful technique first introduced in 1986 as a scanning probe microscopy that allows the topographic analysis of materials with a resolution of nanometers (Binnig et al., 1986). AFM can also be used to probe the mechanical properties of materials, including biopolymers and living cells, and its accurate nanometer control of the sample position allows the study of single molecules (Efremov et al., 2020; Mora et al., 2020). The basic setup of an AFM consists of a probe or tip situated in the border of a cantilever, and a piezoelectric actuator that controls the proximity between sample and tip. Interactions between sample and tip cause the cantilever to bend, and this bending is registered by reflecting a laser beam off the back of the cantilever onto a photodiode. The deflection of the cantilever causes changes in the voltage registered by the photodiode. The relationship between cantilever deflection and changes in the voltage (sensitivity) is established during a previous calibration. Subsequently, this deflection of the cantilever is converted into force after determining the spring constant of the cantilever (Hughes and Dougan 2016; Mora et al., 2020). In mechanical protein unfolding experiments (AFM‐SMFS), the sample, normally a construct that contains several repeats of the protein of interest (POI), is fixed at one end to a surface. Then, the tip and the sample are brought into contact, allowing the protein construct to randomly adhere to the tip (Mora et al., 2020). Two different experimental approaches are possible in this setup. In the first and simpler approach, known as force‐extension, the surface is retracted from the tip (or the tip from the surface) at a constant speed and the force is registered as a function of the increase in the protein extension (Figure 1). This retract causes the protein construct to straighten and then the tensile force of the protein causes the cantilever to bend toward the construct, which is registered as an increment in the force. Further increasing the distance between tip and sample will cause the pulling force to overcome the forces that keep one of the repeats folded, causing the unfolding of that repeat. This shows as a sudden decrease in the force and a subsequent increase in the distance between tip and sample without further increase in force. The change in force is repeated once for each repeat of the protein in the construct, leading to a characteristic saw‐tooth‐like pattern in the force‐versus‐distance trace (Figure 1). In the second experimental approach, called force‐clamp, the protein construct is held at a constant force, and the extension of the protein is registered as a function of the time. In this approach, the unfolding of each protein repeat is seen as a sudden increase in the protein extension with a corresponding dwell time (Mora et al., 2020).

In this primer, we describe a simple method to analyze the mechanical stability and energy landscape of protein unfolding using atomic force microscopy. We discuss the preparation of polyprotein constructs suitable for SMFS with AFM equipment describes the parameters used in our force‐extension SMFS experiments and the models and equations employed in the analysis of the data. As a practical example, we show the results obtained with a construct containing nine repeats of the I27 domain from the muscle protein titin, including the influence of the temperature on the mechanical unfolding parameters, such as the distance to the transition state, the height or energy of the transition state barrier and the spring constant of the protein in the direction of the unfolding.

## 2 MATERIALS AND METHODS

### 2.1 Preparation of protein samples for AFM‐SMFS

The first step in AFM‐SMFS experiments is the preparation of the POI, usually in the form of a polyprotein. A polyprotein is a long polypeptide chain that contains several repeats or domains of a POI (usually from 4 to 8) that unfold independently from each other. When all the repeats are identical, the construct is called a homopolyprotein. In turn, heteropolyproteins consist of repeats of a known protein with the protein of interest intersected. Using a polyprotein provides the benefit of a characteristic and regular unfolding pattern generated by the consecutive unfolding of similar domains. Such pattern helps distinguish the unfolding of the POI from unspecific interactions between protein and surface. In the case of heteropolyproteins, it provides the well‐defined and prototypical unfolding of the protein marker that can be used to compare with the POI (Hughes & Dougan, 2016; Mora et al., 2020; Steward et al., 2002; Yang et al., 2020). The latter approach is especially useful when working with intrinsically disordered proteins (IDPs).

Polyprotein synthesis is usually achieved through cDNA concatenation (Carrion‐Vazquez et al., 2000; Hughes & Dougan, 2016). DNA fragments that contain the specific sequence of the POI and restriction sites in the borders can be sequentially digested with the restriction enzymes and then ligated, eventually generating a sequence that contains all the repeats. For the polyprotein, the following should be taken into account: how to link the polyprotein to the surface (and the tip of the cantilever), the number and order of the repeats inside the construct, and the linker sequences between the domains. Regarding the attachment method, the simplest one consists in the non‐specific adsorption of the polyprotein to gold or mica surfaces (Best et al., 2003; Rief et al., 1997; Rief et al., 1999) and to the tip of the cantilever. To avoid the problem of unspecific interactions, cysteines can be engineered in the part of the polyprotein that should attach to the surface or the cantilever (Yang et al., 2020). The number of repeats should be large enough (between eight and ten) to discern the characteristic unfolding pattern of the POI and reduce surface interactions. However, this number should not be too high in order to avoid the so‐called N‐effect (Zinober et al., 2002), where increasing the number of domains in a polyprotein decreases the mean unfolding force of the domain. For heteropolyproteins, the POI is placed between several copies of a well‐known protein, such as the immunoglobulin‐like domain (I27) of titin protein.

In the present work, we have employed a homopolyprotein containing nine repeats of cardiac titin I27, which is one of the immunoglobulin (Ig) domains present in the giant muscle protein titin (Linke & Grutzner, 2008). The mechanical resistance to unfold this domain is generated by several hydrogen bonds formed between the β‐sandwich strands A and B, and A' and G (Higgins et al., 2006; Linke & Grutzner, 2008; Marszalek et al., 1999; Taniguchi et al., 2008).

For the expression of our titin polyprotein, we used the pEMI91 vector which was a gift from Piotr Marszalek (Addgene plasmid # 74888) (Scholl et al., 2016). The plasmid contains 9 repeats of Titin I27 which are codon shuffled for optimized expression in *Escherichia coli*. The protein was expressed in BL21 DE3 *E. coli* strain and protein production was induced by IPTG at an OD of 0.6 for 4 hours. Cells were centrifuged, resuspended in lysis buffer, ultrasonicated and debris was removed by centrifugation. The protein was then purified by Ni^2+^‐affinity chromatography using imidazole as elution agent. Protein content was monitored by UV absorption at 280 nm. If protein‐containing elutes were checked via SDS PAGE, then proteins were dialyzed to PBS buffer, concentrated, and stored at −80°C prior to use.

### 2.2 AFM‐SMFS experiments

For AFM measurements, freshly evaporated gold surfaces were used. 24 mm round borosilicate glass coverslips were cleaned with ethanol and oxygen plasma, and then coated with 3 nm of chromium and a top layer of 10 nm of gold using a sputter coater. The underlying layer of chromium ensures the stable coating of the surface with the gold.

For the mechanical unfolding measurements, a JPK Nanowizard III (Bruker, Germany) was utilized. A BioCell™ chamber (JPK instruments, Germany) was used to maintain the protein solution at the temperature of choice. Before the experiments, untreated triangular silicon nitride cantilevers with a pyramidal tip (DNP‐S, B, Bruker) were cleaned with ozone/UV, and then calibrated on a clean glass surface covered with PBS, taking into account the temperature of the experiment. The spring constant of the cantilever was calibrated using the thermal fluctuation method (Hutter & Bechhoefer, 1993). The cantilevers had a nominal spring constant of 0.12 N/m, a resonance frequency of 23 kHz in air and a tip radius of 10 nm.

After calibration, a gold‐coated coverslip was placed into the AFM cell, rinsed with PBS, and then the purified polyprotein solution was added on top of the gold surface. To avoid several proteins attaching to the tip at the same time, the protein solution was diluted (final concentration of 100 μg/mL). After 15 min, the surface of the coverslip was rinsed with PBS several times to remove unattached proteins. For the rest of the experiment the chamber was refilled with PBS. A large number of force curves must be registered to obtain a usable number of unfolding events. In this primer, at least 3600 force‐extension curves per pulling speed were recorded at different pulling speeds (400, 800, 1600, 3200, 6400, and 12,800 nm/s). To do that, 9 μm^2^ force maps of the surface covered in proteins were obtained. The area was subdivided in 900 pixels, each pixel consisting in one single measurement, which provided enough separation between measurements (0.1 μm) and therefore decreased the probability of the same polyprotein being picked twice. The tip was approached at the desired speed, maintained in contact with the surface for 1 s with a set force of 2 nN, and then retracted at the same pulling speed. The motion of the piezo was set to 400 nm for the approaching and retracting curves, with an acquisition rate which ranged from 2500 to 80,000 Hz depending on the pulling speed (higher acquisition rates for higher pulling speeds).

The measurements were repeated two different times for each experimental condition, using different cantilevers. To analyze the effects of temperature on the mechanical unfolding, three different temperatures were taken into account 18°C, 23°C, and 37°C.

### 2.3 Data analysis

#### 2.3.1 Filtering the force curves

**FIGURE 2:**
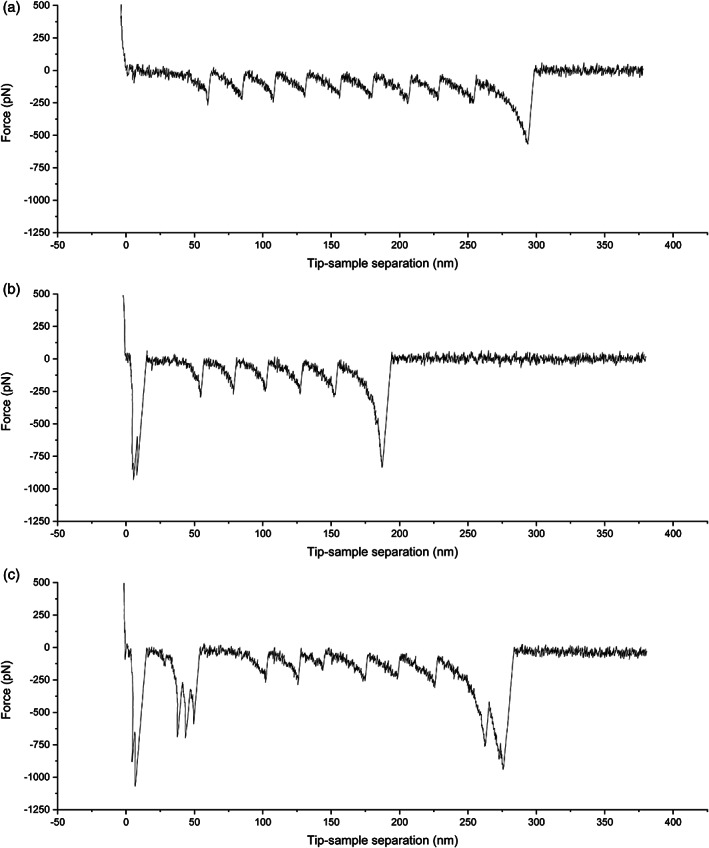
Example curves from protein unfolding SMFS experiments of a construct containing nine repeats of I27. (a) Shows a perfect curve where the unfolding of all nine repeats is registered, showing a regular saw‐tooth pattern. (b) Shows a good curve: The number of unfolding events is less than nine but higher than three, and the initial unspecific adhesion does not affect the pattern. (c) Shows a discarded curve where the pattern is irregular: The distance between unfolding events is not constant.

Before analyzing the unfolding of the POI, one should discard all curves that show bad unfolding patterns. A perfect unfolding curve for a homopolyprotein is shown in Figure 2a. Such curve presents a number of equidistant unfolding events (saw‐tooth pattern) equal to the total number of repeats of the POI present in the construct (Best et al., 2003).

However, this is normally not the case, since the attachment of the protein to the tip occurs randomly at any point in the protein. Therefore, the curve depicted in Figure 2b could be also considered for analysis. Since the first unfolding event can be affected by unspecific tip‐surface interactions, and the last event corresponds to the detachment of the protein and the tip, these peaks are usually discarded for the analysis. Thus, any curve with less than three unfolding events is not used for analysis. Thus, a simple rule to do an initial filtering of the force curves is to discard any curves that show large surface adhesion, irregular patterns (Figure 2c) and low number of unfolding events. Curves showing a number of unfolding events higher than the number of repeats of the polyprotein should also be discarded (they are probably caused by the attachment and unfolding of several constructs at the same time).

#### 2.3.2 Fitting a molecular extension model and obtaining the contour length of the POI

**FIGURE 3:**
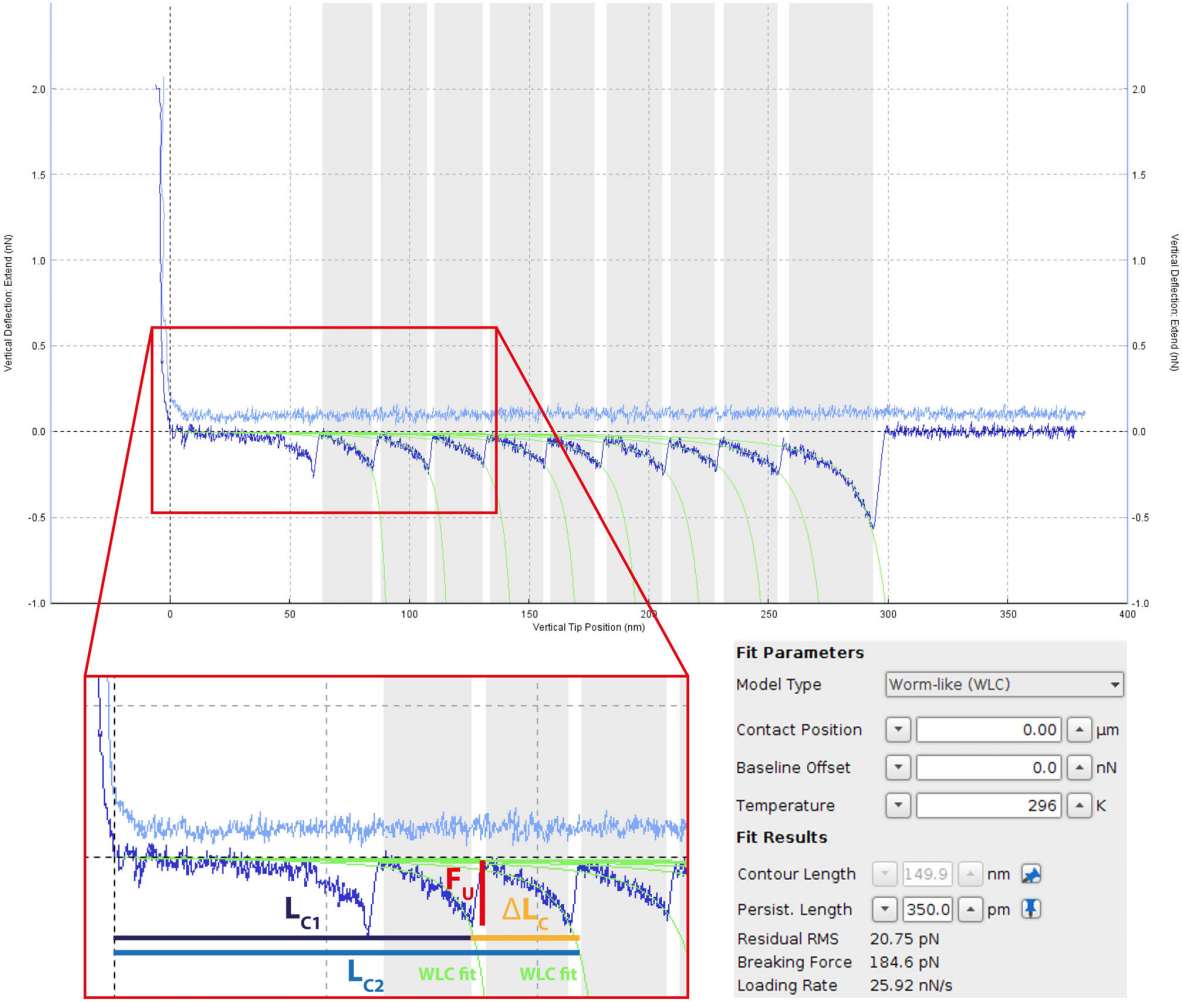
Example of a force‐versus‐extension curve where each unfolding event has been fitted with the WLC model (green lines) using the default software of the JPK III. In the red square a close view of the fitting can be observed. Each unfolding event shows a specific unfolding force ($F_{U}$) and an increase in the contour length (${\Delta L}_{C}$). The last one can be obtained by subtracting the contour length of the previous unfolding event ($L_{C1}$) to the current contour length ($L_{C2}$). Additionally, each unfolding event shows a specific loading rate in the moment of the unfolding, the CLR.

After the selected curves are corrected (i.e., baseline, contact point, and tip‐surface distance) one can obtain the unfolding force ($F_{U}$) and (the increment in) the contour length (${\Delta L}_{C}$) of each unfolding event (Figure 3). While the (*F*_*U*_) can be determined directly, (*ΔL*_*C*_) depends on the distance between peaks (i.e. on the number of amino acids contained inside the repeat). A theoretical estimation of (${\Delta L}_{C}$) can be obtained by multiplying the number of amino acids by its average size (0.365 nm). In order to obtain (${\Delta L}_{C}$) a model of polymer elasticity should be applied such as the worm‐like chain (WLC) (Bustamante et al., 1994; Marko & Siggia, 1995), the freely jointed chain (FJC) (Ortiz & Hadziioannou, 1999), or the freely rotating chain (FRC) (Livadaru et al., 2003).

Here, we used the WLC model, which considers the protein as a deformable chain composed of rigid elements which have a characteristic persistence length. In the absence of force, the polymer remains in a random collapsed conformation. When a force is applied, there is an entropic resistance to elongation. The measured (unfolding) force (F) for each unfolding event is correlated with the contour length (${\Delta L}_{C}$) through the following equation:

(1) $$\frac{\mathrm{Fp}}{k_{B}T}=\frac{z}{L_{C}}+\frac{1}{4{\left(1-\frac{z}{L_{C}}\right)}^{2}}-\frac{1}{4},$$

where (p) is the apparent persistence length (approximated to the average length of an amino acid, 0.35 nm, in our experiments), ($k_{B}$) is the Boltzmann constant, (T) the absolute temperature, and (z) the end‐to‐end length of the molecule. The fitting of each unfolding event (with the JPK software), provides the increment in the contour length (${\Delta L}_{C}$) of each unfolding event by subtracting the contour length of the previous unfolding event (see Figure 3).

For this reason, the first unfolding event and the last must be discarded. (${\Delta L}_{C}$) is characteristic of the POI and therefore can be used to make sure to detect possible artifacts. In heteropolyproteins, (${\Delta L}_{C}$) is also used to distinguish between different proteins.

The JPK software also delivers the values of the total contour length ($L_{C}$), the unfolding force ($F_{U}$) and the critical loading rate (CLR) (Figure 3). The loading rate (r) in the moment of the unfolding is proportional to the pulling speed (v) times the effective stiffness (k) of both the cantilever and the sample. In our case, the evaluated contour length (${\Delta L}_{C}$) of each I27 domain was 27.7 nm, which is in agreement with published values (Carrion‐Vazquez et al., 1999; Muddassir et al., 2018). Alternatively, other softwares can also be used to fit this model (Lamour et al., 2014).

#### 2.3.3 Analyzing the energy landscape of mechanical protein unfolding

**FIGURE 4:**
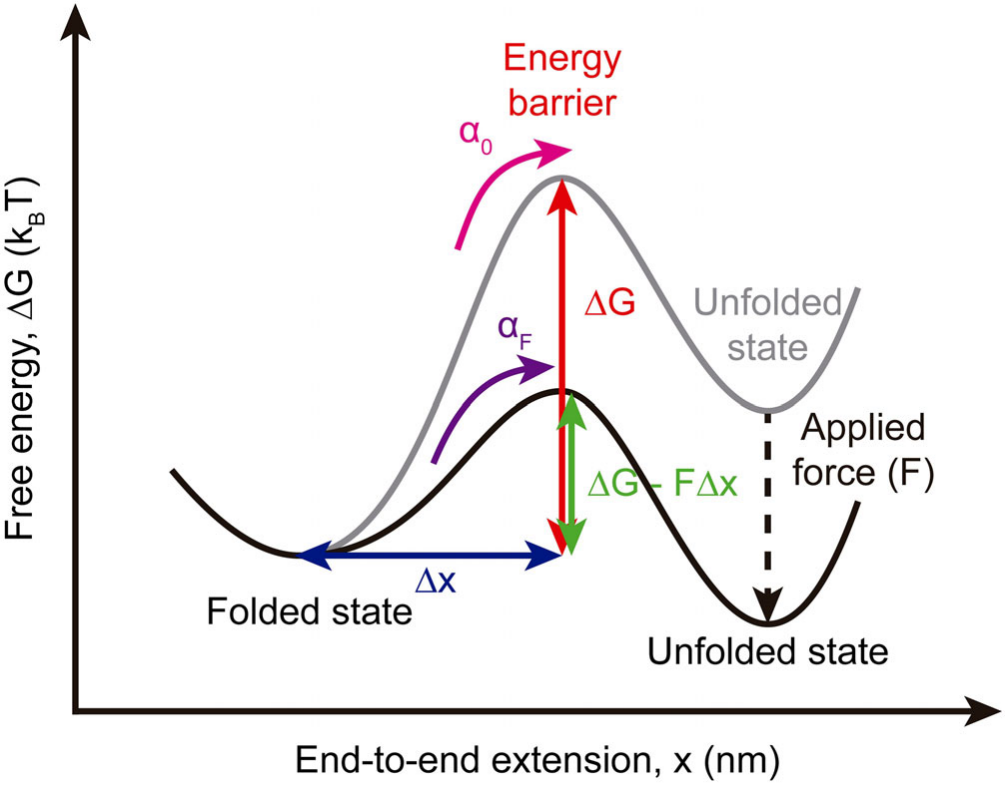
Mechanical energy landscape of a protein with two states. In basal conditions, the folded and unfolded energy states of the protein are separated by an energy barrier of height (∆G). Additionally, the folded state is separated from the energy barrier a certain distance (∆x). The unfolding rate in the absence of force is ($\alpha_{0}$). When an external force (F) is applied a distance (∆x), the energy barrier is lowered from (∆G) to (∆G−F∆x). The energy landscape is therefore tilted toward the unfolded state, and the unfolding rate increases to ($\alpha \left(F\right)$).

The energy landscape of protein unfolding is a high‐dimensional surface that includes all possible conformations that a protein may acquire. In this energy landscape, the protein has the most stable conformation with the lowest free energy (∆G), when it is correctly folded (Hughes & Dougan, 2016; Mora et al., 2020). Since the applied force is directional the mechanical unfolding can be described as a one‐dimensional projection of the energy landscape (Figure 4). To mechanically unfold the protein, the energy barrier must be overcome, being the unfolding rate of the protein in the absence of force ($\alpha_{0}$). When an external force is applied the energy barrier is lowered from (∆G) to (∆G−F∆x) increasing the unfolding rate. Here (∆x) is the distance between the folded state and the energy barrier (distance to the transition state). Various models describe how an external force affects the unfolding rate of the protein and the height of the transition barrier (Dudko et al., 2006; Evans & Ritchie, 1997; Friddle et al., 2012; Hughes & Dougan, 2016; Mora et al., 2020).The Bell‐Evans‐Richie model predicts a linear dependence between the unfolding force and the natural logarithm of the loading rate.

The loading rate (r) depends on the speed that the protein is being pulled or extended

(2) $$r=\frac{\mathrm{dF}}{\mathrm{dt}}=k\times v,$$

where (k) is the effective stiffness (dependent on the cantilever spring constant and the protein stiffness) and (v) the pulling speed.

**FIGURE 5:**
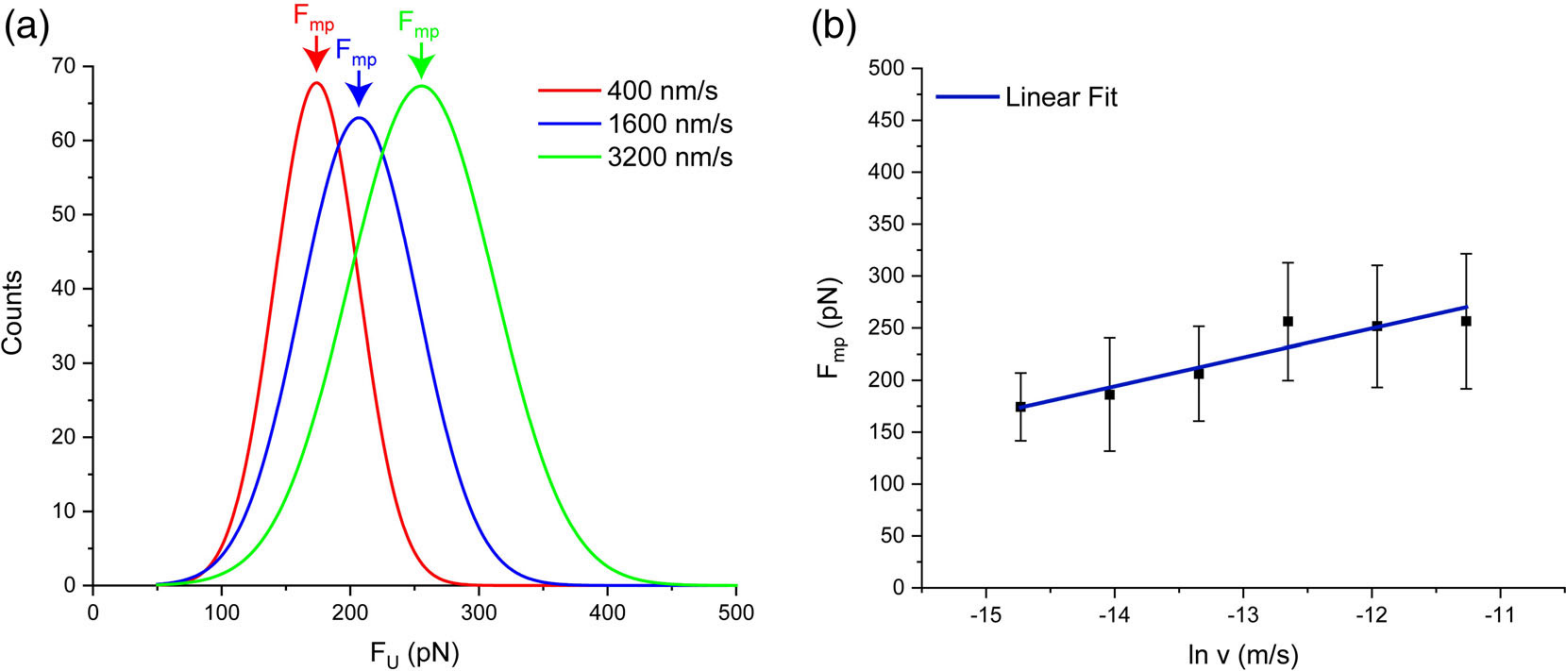
Estimating the parameters of the energy landscape using the bell‐Evans‐Ritchie model. (a) Protein unfolding is a stochastic event, therefore we can plot the distribution of unfolding forces for each specific pulling speed in histograms and determine the most probable unfolding force ($F_{\mathrm{mp}}$). (b) Then, the ($F_{\mathrm{mp}}$) can be plotted versus the natural logarithm of the pulling speed (m/s), and fitted with a linear function. Finally, the slope of the linear fit can be used to determine (∆x), while the intercept relates to ($\alpha_{0}$). Note in (a) the displacement of ($F_{\mathrm{mp}}$) to larger values as the pulling speed increases.

The faster the protein is pulled, the shorter the time available for the protein to stochastically surmount the energy barrier at any given force, and in consequence, higher unfolding forces are observed (Figure 5a). Thus several pulling speeds should be tested. Assuming that the protein unfolds in a two‐state manner and (∆x) does not change with the applied force, the most probable unfolding force is related to the loading rate through

(3) $$F_{\mathrm{mp}}=\frac{k_{B}T}{∆x}\ln\frac{r∆x}{\alpha_{0}k_{B}T}$$

where ($F_{\mathrm{mp}}$) is the most probable unfolding force, ($k_{B}$) is the Boltzmann constant, (T) the absolute temperature, (∆x) the distance between the folded state and the energy barrier, (r) is the loading rate and ($\alpha_{0}$) is the unfolding rate in the absence of force. Replacing (r) in Equation 3 for its value in Equation 2 follows

(4) $$F_{\mathrm{mp}}=\frac{k_{B}T\;}{∆x}\;\ln v+\frac{k_{B}T\;}{∆x}\ln\frac{k∆x}{\alpha_{0}k_{B}T}=\frac{k_{B}T\;}{∆x}\;\ln v+c,$$

where (c) is a constant value. This equation shows a linear relationship of the unfolding force with the logarithm of the pulling speed, where the slope is ($\frac{k_{B}T}{∆x}$) and the intercept is (c). Thus, it is simple to evaluate (∆x) and the unfolding rate at zero force ($\alpha_{0}$) by plotting the most probable unfolding force ($F_{\mathrm{mp}}$) for each pulling speed versus the natural logarithm of the pulling speed (lnv) (Figure 5b). However, during the unfolding process, the loading rate (r) changes due to variations in the effective stiffness (k) because of the nonlinear and linear properties of both the cantilever and the protein (Mora et al., 2020). Nevertheless, although we cannot assume full linearity plotting ($F_{\mathrm{mp}}$ versus lnv) is a good method to estimate the value of (∆x).

**FIGURE 6:**
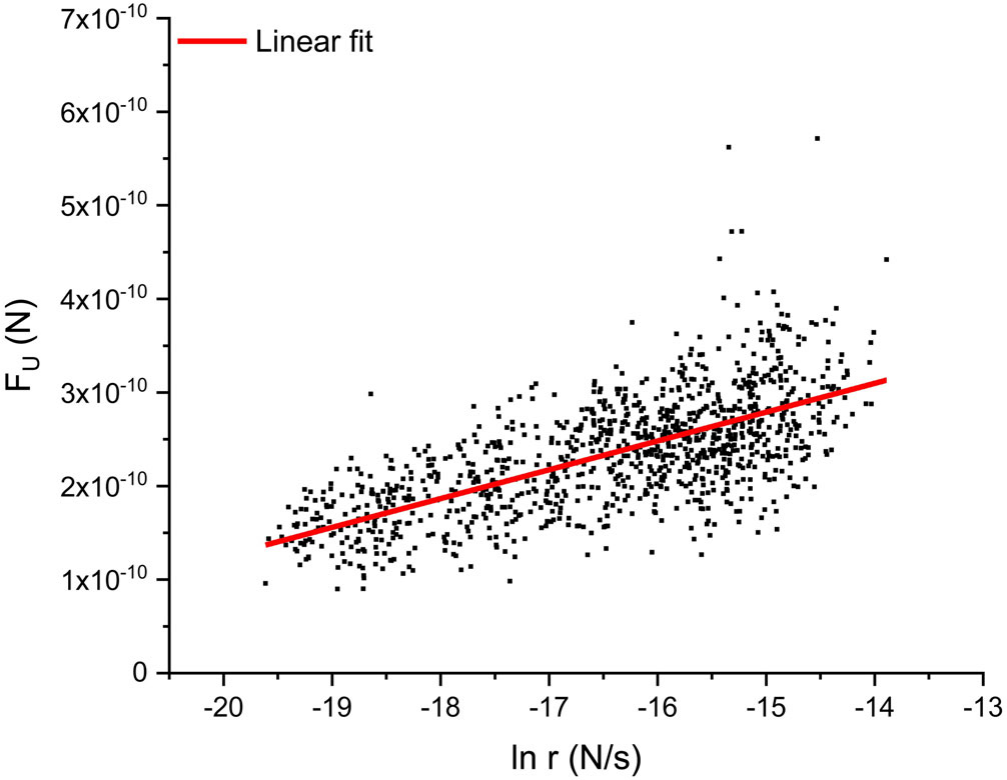
Evaluation of the distance (∆x) and the unfolding rate in the absence of force ($\alpha_{0}$) using the bell‐Evans model. The unfolding force ($F_{U}$) of each unfolding event is plotted versus the natural logarithm of the CLR (loading rate, N/s), and then fitted with a linear function. The slope (S) and intercept (c) of the linear function can be used to calculate (∆x) and ($\alpha_{0}$), respectively.

Monte Carlo (MC) simulations can be used to mimic the stochastic unfolding of the protein using different combinations of (∆x) and ($\alpha_{0}$). The simulations provide force distributions for each pulling speed, which are compared to the experimental force distributions allowing to obtain the values of (∆x) and ($\alpha_{0}$) that best match the experimental data (Best et al., 2002; King et al., 2010; Rief et al., 1998). Here we have also plotted the unfolding force of each unfolding event ($F_{U}$) versus the critical loading rate (CLR), see Figure 6, with (r) being related to the effective stiffness in the moment of unfolding and the pulling speed

(5) $$F_{U}=\frac{k_{B}T\;}{∆x}\;\ln r+c$$

A linear relationship can be observed between ($F_{U}$) and (lnr). Therefore, ($F_{U}$) can be plotted versus the natural logarithm of (r) for all the measured unfolding events for different pulling rates (Figure 6). The slope (S) of that linear function is related to the distance to the transition state (∆x) as follows:

(6) $$∆x=\frac{k_{B}T\;}{S}$$

The intercept (c) of the linear function provides the value of the unfolding rate in the absence of force ($\alpha_{0}$) by

(7) $$c=\frac{k_{B}T\;}{∆x}\ln\frac{∆x}{\alpha_{0}k_{B}T};\alpha_{0}=\frac{1}{e^{c/S}S}$$

One can go further and estimate the height of the energy barrier (∆G) using the Arrhenius equation (Bhattacharya & Ainavarapu, 2018; Schlierf & Rief, 2005)

(8) $$∆G=k_{B}T\;\ln\frac{A}{\alpha_{0}}$$

where (A) is the Arrhenius pre‐factor, estimated to be of the order of 10^7^ s^−1^ in proteins (Lapidus et al., 2000; Schlierf & Rief, 2005; Yang & Gruebele, 2003). However, it should be pointed out that this value varies several orders of magnitude depending on the study, as it is affected by the size of the protein and other parameters (Bhattacharya & Ainavarapu, 2018; Popa et al., 2011; Schlierf & Rief, 2005). The last parameter to consider is the protein spring constant in the pulling direction. The spring constant of the POI can be estimated assuming that the energy landscape follows a parabolic function around the state of minimum energy (Schlierf & Rief, 2005). Thus, the spring constant of the protein (D) can be estimated as follows:

(9) $$D=\frac{2∆G}{∆x^{2}}$$

Changes in the value of (D) can be associated to alterations in the compliance of the protein. The spring constant of the cantilever (ca. 0.06 N/m) should ensure a good signal‐to‐noise ratio.

### 2.4 A practical example: Effects of temperature on the mechanical energy landscape of the titin domain I27

The mechanical stability of proteins is a crucial factor to consider in order understanding their properties and their function. In this section, we present the effects of the temperature on the mechanical unfolding and the energy landscape of I27. Titin acts as an entropic spring in the sarcomeres of muscles (Kellermayer et al., 1997; Li et al., 2002). When the sarcomere is passively stretched (e.g., due to antagonist muscles), the linker regions between Ig domains present in the I‐band straighten, and then the Ig domains unfold and titin extends. After the force is removed, these Ig domains refold, generating work and contributing to the recovery of the resting length of the sarcomere (Freundt & Linke, 2019; Rivas‐Pardo et al., 2016). Thus, investigating the mechanical properties of titin Ig domains contributes to the understanding of the physiological functions of titin in the muscle.

We performed AFM‐SMFS unfolding experiments of our construct for three different temperatures 18°C, 23°C, and 37°C (for details see materials and methods section). As shown in Table 1, the values of (${\Delta L}_{C}$) did not change significantly with the temperature taking a values of about 27.7 nm. This value is in agreement with other reported in the literature (Carrion‐Vazquez et al., 1999; Muddassir et al., 2018; Taniguchi et al., 2008).

**TABLE 1:** (${\Delta L}_{C}$) values of the protein unfolding events for each temperature. N total indicates the total number of individual unfolding events that were quantified, regardless of the pulling speed. The values are indicated as (mean ± standard deviation).

Temperature (°C)	N total	Mean ${\Delta L}_{C}$ (nm)
18	1073	27.8 ± 1.2
23	1206	27.8 ± 1.2
37	1615	27.7 ± 1.2

**FIGURE 7:**
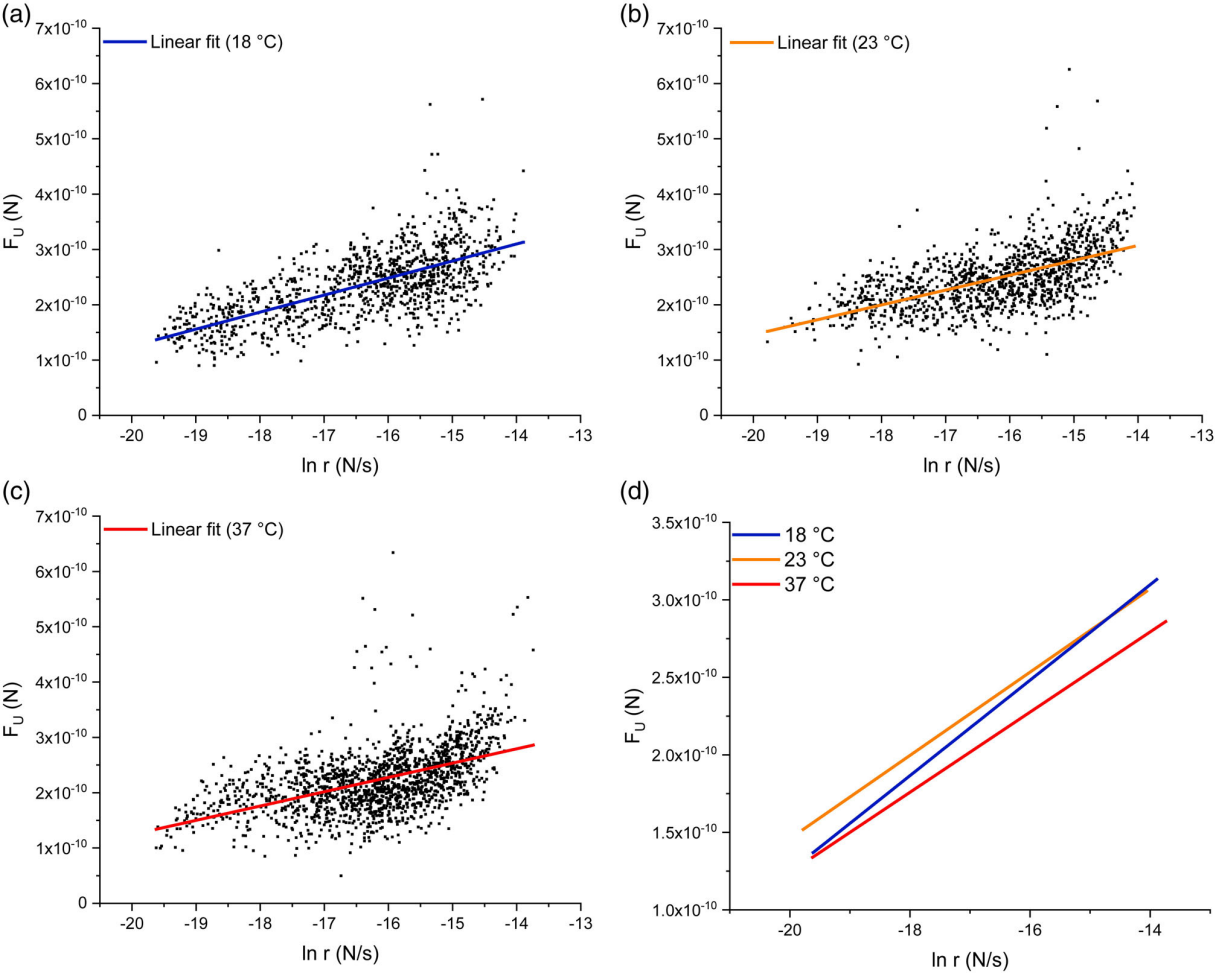
$F_{U}$ versus lnr graphs with the corresponding linear fittings for each set of experimental data. Each individual unfolding event was plotted for temperatures of (a) 18°C, (b) 23°C, and (c) 37°C. graph (d) compares the lineal fittings shown in graphs (a–c)

Subsequently, all the unfolding events for each experimental condition were plotted as ($F_{U}$ versus lnr) as shown in Figure 7. The slope and the intercept were used to evaluate (∆x), ($\alpha_{0}$), (∆G), and (D). All these values are shown in Table 2. It can be observed that (∆x) and (∆G) rose when the temperature was increased, while a decrease in ($\alpha_{0}$) and (D) could be seen. Thermal softening of the protein has also been reported for other protein constructs that include I27 (Taniguchi et al., 2008), and other proteins such as domain 4 of filamin (ddFLN4) (Schlierf & Rief, 2005), and β‐spectrin (Law et al., 2003). Taniguchi et al. (2008) analyzed the mechanical energy landscape of I27 and showed that the force required for the transition of I27 to its intermediate state was not affected by the temperature. They also reported an increase in (∆x) and (∆G) caused by higher temperatures. While the transition to the intermediate state has not been taken into account in our experiments, our results are similar to those shown by Taniguchi et al. (2008). Indeed, the spring constant of the protein in the direction of pulling (D) also decreased with higher temperatures as expected.

**TABLE 2:** Effect of temperature on the parameters of the mechanical energy landscape of I27. (∆x) is the distance between the folded state and the energy barrier, ($\alpha_{0}$) is the unfolding rate in the absence of force, (∆G) is the height of the energy barrier and (D) is the spring constant of the protein in the direction of the pulling. The values are indicated as (mean ± standard deviation).

Temperature (°C)	∆x (nm)	$\alpha_{0}$ (s^−1^)	∆G (k_B_T)	D (N/m)
18	0.13 ± 0.01	1.17 ± 0.28	15.98 ± 0.24	7.54 ± 0.43
23	0.15 ± 0.01	0.34 ± 0.11	17.22 ± 0.34	6.08 ± 0.38
37	0.17 ± 0.01	0.67 ± 0.21	16.55 ± 0.32	5.17 ± 0.34

Since the unfolding of I27 depends on the disruption of the hydrogen bonds between strands A' and G, the effect of temperature in the studied parameters could be a consequence of the destabilization of such bonds. Experiments with mutant proteins where some residues important for the establishment of hydrogen bonds are replaced have shown an increase in (∆x) and (∆G) (Li et al., 2000).

## 3 CONCLUSIONS

In this primer, we have presented the first concepts and experimental steps that a researcher has to take into account when studying the mechanical unfolding of biopolymers with AFM. With this method it is easy to determine accurately the unfolding force and the possible transition states, and with some error, the unfolding constant at zero force. It is also briefly reported how to obtain the strength of the interaction (analogous to a spring constant) when the protein is folded (free energy minimum).

Thus, single‐molecule experiments with AFM provide valuable information to study the mechanical stability and the energy landscape of biopolymers, because it targets specifically those bonds that keep the biopolymer folded. The combined use of AFM with optical tweezers permits to elucidate the response of the biopolymer to force at slow rates that the AFM cannot achieve (this will deliver more accurate values of the unfolding rate at zero force). The combination of AFM, optical tweezers, nuclear magnetic resonance, cd spectroscopy, fluorescence spectroscopy, and classical bulk denaturation experiments provide a compact description of the structure, stability and function of biopolymers.

## CONFLICTS OF INTERESTS

The authors declare no conflict of interest.

## AUTHOR CONTRIBUTIONS

Juan Carlos Gil‐Redondo, Andreas Weber, José L. Toca‐Herrera: Conceptualization. Juan Carlos Gil‐Redondo, Andreas Weber: Experiments. Juan Carlos Gil‐Redondo, Andreas Weber, José L. Toca‐Herrera: Formal analysis. Juan Carlos Gil‐Redondo, Andreas Weber, José L. Toca‐Herrera: Methodology. Andreas Weber, José L. Toca‐Herrera: Supervision. All authors contributed to writing, editing and reviewing the manuscript.

## Data Availability

Data supporting can be accessed upon direct request to the authors.
